# Inhaled corticosteroids in ventilated preterm neonates: a non-randomized dose-ranging study

**DOI:** 10.1186/s12887-018-1134-7

**Published:** 2018-05-07

**Authors:** Kamini Raghuram, Michael Dunn, Krista Jangaard, Maureen Reilly, Elizabeth Asztalos, Edmond Kelly, Michael Vincer, Vibhuti Shah

**Affiliations:** 1 Department of Paediatrics, University of Toronto, Toronto, ON Canada; 2 Department of Newborn Medicine and Developmental Medicine, Sunnybrook Health Sciences Centre, Toronto, ON Canada; 3 Department of Paediatrics, Izaak Walton Killam (IWK) Health Centre, Dalhousie University, Halifax, NS Canada; 4 Department of Paediatrics, Mount Sinai Hospital, 600 University Avenue, Rm 19-231, Toronto, ON M5G 1X5 Canada

**Keywords:** Infant-newborn, Preterm, Inhaled steroid, Metered dose inhaler, Bronchopulmonary dysplasia

## Abstract

**Background:**

Inhaled corticosteroids (ICS) offer targeted treatment for bronchopulmonary dysplasia (BPD) with minimal systemic effects compared to systemic steroids. However, dosing of ICS in the management of infants at high-risk of developing BPD is not well established. The objective of this study was to determine an effective dose of ICS for the treatment of ventilator-dependent infants to facilitate extubation or reduce fractional inspired oxygen concentration.

**Methods:**

Forty-one infants born at < 32 weeks gestational age (GA) or < 1250 g who were ventilator-dependent at 10–28 days postnatal age were included. A non-randomized dose-ranging trial was performed using aerosolized inhaled beclomethasone with hydrofluoralkane propellant (HFA-BDP). Four dosing groups (200, 400, 600 and 800 μg twice daily for 1 week) with 11, 11, 10 and 9 infants in each group, respectively, were studied. The primary outcome was therapeutic efficacy (successful extubation or reduction in FiO_2_ of > 75% from baseline) in ≥60% of infants in the group. Oxygen requirements, complications and long-term neurodevelopmental outcomes were also assessed.

**Results:**

The median age at enrollment was 22 (10–28) postnatal days. The primary outcome, therapeutic efficacy as defined above, was not achieved in any group. However, there was a significant reduction in post-treatment FiO_2_ at a dose of 800 μg bid. No obvious trends were seen in long-term neurodevelopmental outcomes.

**Conclusions:**

Therapeutic efficacy was not achieved with all studied doses of ICS. A significant reduction in oxygen requirements was noted in ventilator-dependent preterm infants at 10–28 days of age when given 800 μg of HFA-BDP bid. Larger randomized trials of ICS are required to determine efficacy for the management of infants at high-risk for development of BPD.

**Trial registration:**

This clinical trial was registered retrospectively on clinicaltrials.gov. The registration number is NCT03503994.

## Background

While many short-term morbidities associated with extreme prematurity have declined over the last two decades, the incidence of bronchopulmonary dysplasia (BPD) has increased to a rate of approximately 45% in neonates < 28 weeks gestational age (GA) and birth weight (BW) < 1500 g [1, 2]. Neonates with BPD are at increased risk for adverse short-and long-term neurodevelopmental and respiratory outcomes that often persist into adulthood [3, 4].

There is a growing body of pathological and biochemical evidence that implicates inflammation in its pathogenesis [5–7]. This is further supported by randomized controlled trials (RCTs) that demonstrate the efficacy of systemic corticosteroids in facilitating extubation and reducing BPD [8, 9]. However, several short- and long-term adverse effects associated with the use of systemic corticosteroids have been described [8–10], the most concerning of which is their effect on neurodevelopment, specifically an increased rate of cerebral palsy (CP) [11].

Inhaled corticosteroids (ICS) are an attractive alternative to systemic steroids because of these concerns. Earlier systematic reviews had not found any benefit in using ICS for the prevention or treatment of BPD [12]. However, a recent systematic review showed a significant reduction in death or BPD at 36 weeks’ corrected GA (CGA) (risk ratio = 0.86, 95% confidence interval 0.75, 0.99), BPD (RR = 0.77, 95% CI 0.65, 0.91), and use of systemic steroids (RR = 0.87, 95% CI 0.76, 0.98) in infants treated with ICS [13].

Despite growing evidence of the effectiveness of ICS for BPD, uncertainty remains over treatment timing, effective dose, and long-term effects. There is also variation in the delivery systems used for delivery of ICS. These concerns continue to be echoed in a recent review by Nelin et al. [14]. Given that the long-term neurodevelopmental impact of ICS were unknown at the time of this study and many infants are able to wean from ventilation without steroids, we conducted an escalating-dose ranging study of late ICS (i.e. administered after the first week of life) delivered by a metered dose inhaler (MDI) utilizing a specially designed valved delivery system to determine the minimum effective dose necessary to achieve extubation or reduction in oxygen requirements and the long-term neurodevelopmental impact of increasing doses of ICS.

## Methods

The study was conducted in the NICUs of Mount Sinai Hospital (MSH), and Sunnybrook Health Sciences Centre (SHSC), Toronto, Ontario and Izaak Walton Killam (IWK) Health Centre, Halifax, Nova Scotia, Canada from March 2002 to October 2006. The Mount Sinai Hospital Research Ethics Board, the Sunnybrook Health Sciences Centre Research Ethics Board and the Izaak Walton Killam Research Ethics Board approved this study.

### Study population

Neonates with BW < 1250 g and GA < 32 weeks with need for mechanical ventilation (defined as conventional ventilation with a rate > 15 breaths/min or high frequency oscillatory ventilation) and fractional inspired oxygen concentration (FiO_2_) of > 0.30 but < 0.60), postnatal age of 10–28 days and stable ventilator requirements over the 48–72 h prior to enrollment were included. Baseline demographics, including GA, BW, Apgar scores at 1 and 5 min of life, and presence of respiratory distress syndrome (FiO_2_ > 0.30 or significant work of breathing and/or the need for surfactant therapy) were collected (Table 1). Neonates with actual or suspected sepsis, congenital cardiorespiratory malformation, patent ductus arteriosus, any stage of necrotizing enterocolitis (NEC), gastrointestinal hemorrhage, perforation or treatment with systemic dexamethasone were excluded. These criteria were chosen in order to avoid confounding with other coexisting conditions that may result in inability to wean ventilation or necessitating high FiO_2_.

**Table 1:** Demographic characteristics of the study population

Variable^a^	200 μg bid (*n* = 11)	400 μg bid (*n* = 11)	600 μg bid (*n* = 10)	800 μg bid (*n* = 9)	*p*-value
Gestational age (weeks), mean (SD)	25.7 (1.3)	25.6 (1.2)	25.7 (1.6)	25.4 (1.6)	0.97
Birth weight (grams), mean (SD)	788 (224)	802 (231)	749 (209)	754 (188)	0.91
Apgar score at 1 min, median (range)	5 (1, 7)	6 (2, 8)	5 (1,9)	3 (2, 9)	0.33
Apgar score at 5 min, mean (range)	8 (4, 9)	8 (4, 9)	8 (5, 10)	7 (3, 8)	0.81
Respiratory distress syndrome, %	100	100	90	100	0.37

^a^ bid = twice a day, *μg* microgram, *n* number, *SD* Standard deviation

### Drug regimen and delivery

Groups of neonates were treated with escalating doses (200, 400, 600 and 800 μg twice daily [15]) of hydrofluoralkane beclomethasone dipropionate (HFA-BDP) until efficacy or significant side effects were observed. If the baby was extubated within 7 days after dosing had commenced, administration of ICS was discontinued at that point. Ventilatory settings were temporarily adjusted to achieve an expiratory tidal volume of 7 ml/kg (based on current weight) with a respiratory rate of 30 breaths/minute and positive end expiratory pressure (PEEP) of 5–8 cm H_2_O. Arterial oxygen saturation was maintained within the target range of 88–92%. Following drug administration, the delivery system was removed and baby returned to the previous ventilatory settings. Infants who were on HFO ventilation were transferred onto conventional ventilation for the treatment. The assessors were not aware of the dosing allocations.

### Adverse events

Adverse events including hypertension (defined as BP higher than 2 standard deviations [SD] above the mean for the infant’s gestational and postnatal age), [16] hyperglycemia (defined as a blood glucose > 10 μmol/L), impaired growth (defined as weight loss or head circumference decreases crossing percentile lines on the sex-specific Fenton chart), [17] sepsis, evidence of feeding intolerance, NEC or intestinal perforation, and oropharyngeal candidiasis were recorded pre- and post-treatment. The Safety Committee reviewed adverse events in a particular dosing group before providing approval for recruitment of patients into the subsequent dosing group or recommendation to stop the trial.

### Primary outcome

The primary outcome was therapeutic efficacy, defined by successful extubation or reduction in FiO_2_ of > 75% from the baseline within the one-week study period in ≥ 6 out of 10 infants in each group set a priori. This was based on expert consensus and on previous similar studies [18, 19]. Pre- and post-treatment FiO_2_ was determined by calculating the mean of the FiO_2_ requirement over the preceding 48 h and over the final 48 h of the study period, respectively. If a patient was extubated successfully, the post-treatment FiO_2_ was determined by calculating the mean of the FiO_2_ measured over the last 48 h while intubated. A standard weaning protocol was used prior to extubation from the ventilator. Once the neonate had achieved a MAP of 8 cm H_2_O, ventilator rate of 10–12 breaths/minute and FiO_2_ < 0.30, the neonate was to be extubated, at the discretion of the clinical team. Extubation was considered successful if the infant did not require assisted ventilation in the following 48 h.

### Secondary outcomes

Secondary outcomes included long-term neurodevelopmental outcome. Long-term motor and cognitive function were assessed using validated tests administered by trained personnel in the respective Neonatal Follow-up Clinics. The use of specific tests was not mandated but levels of impairment were aligned. Standardized developmental testing was performed at 18–36 months CA and the assessors were not aware of the dosing allocation. Motor impairment was defined as the presence or absence of CP and, if present, was assessed for severity by Gross Motor Functional Classification Scale- Extended and Revised (GMFCS-E&R); severe CP (non-ambulatory) was defined as GMFCS ≥3 [20]. Severe cognitive impairment was defined as a Bayley Scales of Infant Development-2nd Edition (BSID-II) Mental Developmental Index of < 70, a Bayley Scales of Infant Development 3rd Edition (BSID-III) cognitive score of < 85, [21] or scores below 2 standard deviations from the mean on the Differential Abilities Scales (DAS), a comprehensive test to assess cognitive abilities important to learning [22] or the Clinical Adaptive Test/Clinical Linguistic and Auditory Milestone Scale (CAT/CLAMS), a screening tool for children suspected of having developmental concerns [23]. Mild to moderate cognitive delay was defined as BSID-II Mental Developmental Index score 70–85 or BSID-III cognitive score of 85–100.

### Statistical analysis

Continuous data were analyzed using analysis of variance while categorical data were analyzed using chi-square test. A *p* value of < 0.05 was considered statistically significant. The frequency of the neurodevelopmental characteristics above were calculated for each dosage group. Descriptive statistics are used to present the data for neurodevelopmental outcome given the small numbers and varying tests used. A sample size of 10 neonates per dosing group was chosen, similar to other investigational phase II clinical trials [24]. A formal sample size calculation based on a power of 80% and ɑ error threshold of 0.0125 (corrected for 4 dosing groups) for the primary outcome of extubation or reduction in FiO_2_ in 2/3 of the infants in each group based on previous work done by Ohlsson et al. with dexamethasone yielded a sample size of 8 per dosing group [25]. Accounting for 20% attrition, the total sample size for each group was determined to be 10. When the total sample size for each dosing level was met, data were reviewed by a Safety Committee. If no concerns were identified, approval to proceed to recruitment of the next dosing group was given. All neonates in each group were treated with the same dose of ICS.

## Results

A total of 41 subjects were recruited for the study - 11, 11, 10 and 9 infants were enrolled in the 200 μg, 400 μg, 600 μg, and 800 μg bid groups, respectively. As different centers were recruiting simultaneously, 41 subjects instead of the planned 40 were recruited. In the last dosage group, once 9 patients were recruited, it was apparent that the group was not going to achieve efficacy based on the criteria defined and hence recruitment was stopped.

Table 1 shows the baseline demographic characteristics for each dosage group. No significant differences were noted for GA, BW or Apgar scores at 1 and 5 min of life. The mean GA of participating infants was 25 weeks with a mean BW of 775 g.

Table 2 shows the therapeutic efficacy and FiO_2_ requirements prior to and after treatment with inhaled HFA-BDP. The age of commencement of therapy varied from 10 to 28 days with a median of 22 postnatal days across dosage groups. Therapeutic efficacy was not achieved in any group. There was, however a significant reduction in post-treatment FiO_2_ to 0.30 in the group of neonates receiving 800 μg bid of inhaled HFA-BDP while pre-treatment FiO_2_ did not differ.

**Table 2:** Therapeutic efficacy and FiO_2_ requirements at varying doses of HFA-BDP^a^

Variable^a^	200 μg bid (*n* = 11)	400 μg bid (*n* = 11)	600 μg bid (*n* = 10)	800 μg bid (*n* = 9)	*p*-value
Age at commencement of therapy (days), median (range)	21 (12, 27)	26 (10, 28)	22.5 (14, 26)	22 (12, 28)	0.76
Pre-treatment FiO_2_, mean (SD)	0.40 (0.086)	0.39 (0.039)	0.40 (0.10)	0.37 (0.044)	0.78
Post-treatment FiO_2_, mean (SD)	0.40 (0.074)	0.33 (0.071)	0.32 (0.075)	0.30 (0.064)	0.02
Therapeutic efficacy	2/11	2/11	3/10	3/9	0.80

^**a**^*HFA-BDP* Hydrofluoralkane beclomethasone dipropionate, *bid* twice a day, *FiO*_*2*_ Fraction of inspired oxygen, *μg* microgram, *n* number, *SD* Standard deviation

Table 3 shows the neurodevelopmental presentations of enrolled infants. Four infants were lost to follow-up. Three infants were diagnosed with CP and all were ambulatory with GMFCS I. Cognitive function was normal in all infants except one each in the 200 μg and 400 μg groups and two infants in the 800 μg group. Additionally, 2 infants in the 400 μg and 1 infant in the 800 μg group were unable to undergo formal testing. Informal clinical assessment determined that one was normal and two had mild cognitive impairment. There were no obvious trends seen in assessment scores at escalating doses of ICS.

**Table 3:** Neonatal neurodevelopmental outcomes following escalating doses HFA-BDP^a^

Dose	Total Number Assessed	Cerebral palsy, n (%)	Normal or mild cognitive impairment, n (%)	Severe cognitive impairment, n (%)
200 μg bid (*n* = 11)	10	1/10 (10)	9 (90)	1 (10)
400 μg bid (*n* = 11)	10	1/10 (10)	9 (90)	1 (10)
600 μg bid (*n* = 10)	9	0/9 (0)	9 (100)	0 (0)
800 μg bid (*n* = 9)	8	1/8 (12.5)	6 (75)	2 (25)

^a^*HFA-BDP* Hydrofluoralkane beclomethasone dipropionate, *bid* twice a day, *μg* microgram, *n* number, *%* percentage

One infant in the 800 μg group was noted to have hyperglycemia, but required no intervention. There were no other adverse events noted.

## Discussion

Therapeutic efficacy as defined in the protocol was not reached. However, ICS administered to ventilator dependent preterm infants between 10 and 28 days of age at a dose of 800 μg bid resulted in a significant reduction in oxygen requirements after only 1 week of treatment. Thus, this study may provide a starting effective dose of HFA-BDP that can be utilized for future clinical trials. In comparison, Bassler et al. used inhaled budesonide at a dose of 400 μg bid using a very similar delivery system [26]. Previous pharmacokinetic studies indicate that budesonide is approximately 1.6 times more potent than beclomethasone [27]. Thus, at doses equivalent to approximately 500 μg bid of inhaled budesonide, reductions in FiO_2_ requirements were seen. Given that the study was not powered to detect a reduction in FiO_2_, these results should be interpreted with caution.

Based on limited data, even though it appears that two infants in the 800 μg group developed severe cognitive delay, no definitive conclusions can be made regarding neurodevelopmental impact. A dose-dependent trend in cognitive function was not observed and no difference in the motor function was noted.

The use of ICS for the prevention of BPD for infants born < 28 weeks GA has been studied in a number of RCTs and systematic reviews [12, 13, 26, 28]. In a recent randomized study with 441 patients randomized to budesonide and 422 patients randomized to placebo, Bassler et al. [26] investigated the use of early inhaled budesonide within the first 24 h of life. In this study, a reduction in BPD was noted but with a trend toward increased mortality. On the other hand, Nakamura et al. [28] studied 107 infants treated early with fluticasone propionate at a dose of 100 μg and 104 patients treated with placebo and demonstrated no difference in the incidence of death or oxygen dependency at discharge. However, a significant reduction in death or oxygen dependence at discharge was noted for infants between 24 and 26 weeks GA or with chorioamnionitis suggesting that ICS treatment may benefit certain high-risk subpopulations. When these two large studies were included in meta-analyses, ICS used for prevention or treatment of BPD significantly reduced the incidence of BPD and death or BPD [13, 26]. Long-term follow-up from the study by Bassler et al. is pending.

Inhaled corticosteroids used after 7 postnatal days have also been studied. A systematic review of these studies revealed no significant reduction in death or BPD, although the drug types, dosing regimens and delivery systems varied widely [29]. The sample sizes ranged from 19 to 86 infants randomized to either ICS or placebo and the total population included in the systematic review for the primary outcome of death or BPD at 36 weeks PMA was 232. There was a trend towards less use of systemic corticosteroids. In addition, a recent review found that despite small numbers in the included trials, many showed acute changes in pulmonary mechanics following administration of ICS to chronically ventilated preterm infants with BPD [14]. Two of the studies included used inhaled beclomethasone and showed higher rates of successful extubation, increased dynamic airway compliance and reduced airway resistance in oxygen-dependent preterm infants [29]. Neither study showed differences in BPD or mortality. These results are consistent with the findings in our study. However, studies using beclomethasone have been small to date.

**Fig. 1:**
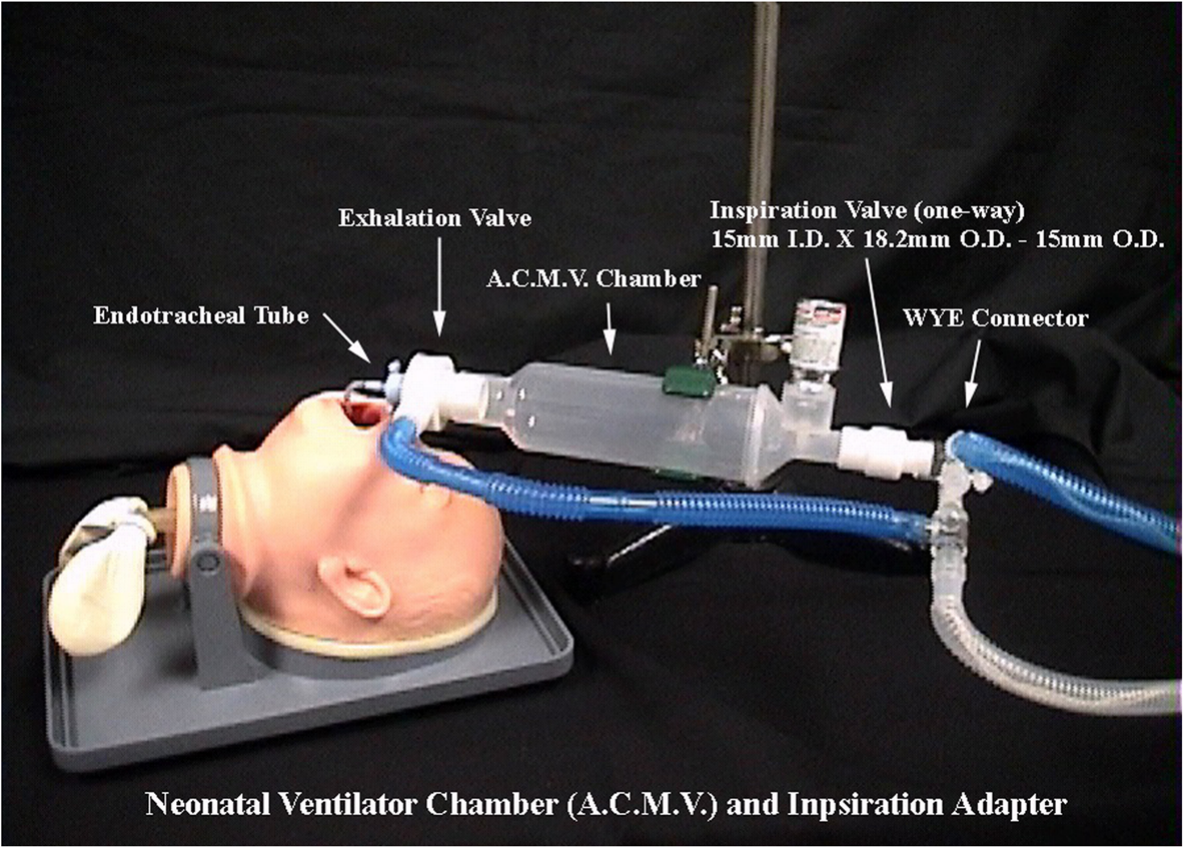
Neonatal Delivery System. Aerosolized HFA-BDP was administered using this specifically designed neonatal aerosol delivery system, including an MDI attached to a valved aerochamber (A.C.M.V). The device was inserted between the endotracheal tube and the WYE-connector of the ventilator circuit. This device is the prototype of the Aerochamber Mini^R^ setup

Our delivery system included an MDI with a propellant that resulted in an aerosol with ideal particle size to enhance deposition. We also used a specifically designed spacer that employed a valve system to improve aerosol delivery. Aerosolized HFA-BDP was administered using a specifically designed neonatal aerosol delivery system (Fig. 1) which included an MDI attached to a valved aerochamber. The device was inserted between the endotracheal tube and the WYE-connector of the ventilator circuit. This device is the prototype of the Aerochamber Mini^R^ used in the study by Bassler et al. [26]. In addition, hydrofluoralkane propellant has been found to allow generation of smaller sized particles and more uniform deposition compared to chloroflurocarbon (CFC) propellant [30]. Despite the improved characteristics of HFA-BDP, overall deposition was estimated to be only 1–2% of the administered dose based on unpublished performance studies (personal communication, M. Dolovich). Thus, therapeutic efficacy may not have been achieved due to poor deposition. On the other hand, studies have shown that MDI delivery is more efficient at ICS delivery than jet nebulizers [31, 32]. This may explain why some studies of ICS did not show significant reductions in oxygen requirements [15]. In addition, while older MDI systems had used CFC propellants, HFA propellant was used in this study and has been found to allow generation of smaller sized particles and more uniform deposition [30]. Thus, more appropriate drug delivery may explain why oxygen requirements were reduced in our study.

Long-term outcomes of infants treated with prolonged courses of ICS are lacking. Nakamura et al. [28] did not demonstrate any significant differences in NDI or CP. In our study, it is reassuring that despite high doses of beclomethasone, there were no obvious differences in long-term neurodevelopmental outcomes. There was actually a trend toward higher rates of mild to moderate impairment in infants receiving lower doses of ICS, indicating that BPD itself may contribute more to poor neurodevelopment.

The strength of this study was its use of escalating doses to determine a pattern of response and the drug delivery system studied. The limitations include the small sample size, and lack of randomization. In addition, patients with PDA were excluded from the study, which limits in generalizability. The other limitation is the delay in publication as a result of individual and system challenges. Since the study period, delivery room practices have evolved to increasing use of early non-invasive ventilation and has resulted in decreased use of prophylactic surfactant with some evidence that these strategies may reduce exposure to invasive ventilation and eventually, chronic lung disease [33–37]. NICU practices have also changed, including less use of systemic steroids, [1] universal, early use of caffeine [38] and increased use of non-invasive modes of ventilation early. However, despite all these changes, the rates of BPD have not improved [1, 2]. As we move toward resuscitating smaller and more preterm infants, [39] BPD will likely remain a significant issue in NICU practice and safe, less invasive, effective therapies are imperative to reducing the morbidity associated with extreme prematurity. Thus, it is our view that ICS may provide a safe and reasonable alternative to systemic steroids in infants who are ventilator-dependent in their 2nd and 3rd week of life and that a larger randomized trial of late inhaled steroids for these children is warranted. In addition, this study provides evidence that the in-line MDI system is an effective way to deliver ICS and that this drug delivery system should be further developed to specifically support ventilated and non-ventilated neonates.

## Conclusions

Treatment with ICS administered using a specially designed system appears to reduce the oxygen requirements in preterm neonates with early BPD with minimal impact on long-term neurodevelopment. Further research into the mechanism of action and appropriate treatment regimens is warranted. Larger studies that examine their impact on long-term neurodevelopment are also warranted.

## Abbreviations

BPD: Bronchopulmonary dysplasia
BSID-II/III: Bayley scales of infant development mental development index
BW: Birth weight
CAT/CLAMS: The Clinical Adaptive Test/Clinical Linguistic and Auditory Milestone Scale
CFC: Chloroflurocarbon
CGA: Corrected gestational age
CP: Cerebral palsy
DAS: Differential Abilities Scales
FiO_2_: Fraction of inspired oxygen
GA: Gestational age
GMFCS: Gross Motor Function Classification Scale
HFA-BDP: Hydrofluoralkane beclomethasone dipropionate
ICS: Inhaled corticosteroids
MAP: Mean airway pressure
MDI: Metered dose inhaler
NDI: Neurodevelopmental impairment
NEC: Necrotizing enterocolitis
PEEP: Positive end-expiratory pressure
RCT: Randomized controlled trial
